# Supplementary material for: MetaRibo-Seq measures translation in microbiomes
Source: Nat Commun. 2020 Jun 29;11:3268. doi: 10.1038/s41467-020-17081-z (PMC7324362; doi:10.1038/s41467-020-17081-z)
Supplement: Supplementary file 10 — Supplementary Data 7 [file 41467_2020_17081_MOESM10_ESM.zip › File2/Confidence_High_Taxonomy/188581_out.krona.html]

Javascript must be enabled to view this page.

members
magnitude
magnitudeUnassigned
count
unassigned
taxon
rank

188581\_out

3

superkingdom
2
3

phylum
1239
3

class
91061
3

186826
order
3

1300
family
3

3
genus
1301


SRS057931\_contig\_number\_1737
1
1318
species

1

SRS051791\_contig\_number\_12525
species
1203590

1305
species

SRS076821\_contig\_number\_contig-100\_971.151487
1
